# Launch of an Innovative Air Pollutant Sampler up to 27,000 Metres Using a Stratospheric Balloon

**DOI:** 10.1007/s42496-023-00151-y

**Published:** 2023-03-09

**Authors:** Federico Toson, Mauro Pulice, Marco Furiato, Matilde Pavan, Simone Sandon, Dumitrita Sandu, Giovanni Righi

**Affiliations:** University of Padova, Via VIII Febbraio 2, Padua, Italy

**Keywords:** Atmospheric pollution analysis, Innovative air sampler, VOCs sampling in the stratosphere, PM research at different altitudes

## Abstract

Air pollution, besides being one of the leading causes of death worldwide, remains one of the most controversial topics in environmental monitoring. The current state of the art refers to remote satellite analysis and static ground-level technologies. The O-ZONE project has set itself the objective of bridging this technological gap using dynamic in situ analysis using compact, inexpensive and reusable samplers that can be integrated onboard stratospheric balloons and drones. The prototype, therefore, consists of a pneumatic system, a set of filters and a sampling bag. Thanks to this architecture, it is possible to sample atmospheric air at different altitudes. After the flight, the samples collected are analysed using chromatographic techniques to provide a picture of the various air layers. On 30 September 2021, the fully autonomous payload successfully flew in Kiruna (Swedish Lapland) aboard BEXUS 30, the stratospheric balloon made available by the promoters of the “hands-on” project of the same name, SNSA (Swedish National Space Agency), DLR (Deutsches Zentrum für Luft- und Raumfahrt) and ESA (European Space Agency). In this paper, the technical specifications of the device are described, with a focus on the sampling system; we then highlight the results obtained by the filters that, at different altitudes, collected stratospheric pollutants such as VOCs and, in the first layers of the atmosphere, PM. In conclusion, an interpretation of the results is provided to better understand the possible future uses of the prototype.

## Introduction

Air pollution, besides being one of the leading causes of death worldwide, remains one of the most controversial topics in environmental monitoring. The causes of climate change are primarily attributable to anthropogenic activities, such as industry, agriculture, intensive livestock farming, transport and an increasing difficulty in disposing of greenhouse gases because of deforestation, which has worsened ecosystems with a consequent deterioration in air quality [1]. Humans, like many other animal species, are also at risk due to the rapid temperature change but mostly because the decreasing quality of the air they breathe may lead to respiratory complications followed, in many cases, by death. The presence of fine dust, harmful combustion products, such as VOCs (Volatile Organic Compounds), CO_2_ and methane (CH_4_) and other greenhouse gases, disrupts the atmospheric composition, increasing global temperature. Moreover, the excessive presence of CFCs (ChloroFluoroCarbons) causes the depletion of the thin layer of Ozone (O_3_) protection against the sun’s UV rays [2, 3].

It is therefore essential to not only define methods of intervention to take care of the situation but also to optimise environmental monitoring processes by refining the technologies available. This willingness to find alternative and more refined monitoring strategies influences the process that regulates the emission of air pollutants and tightens the penalties for those whose environmental damage is considerable, as stated in the Montreal agreement [4].

In recent years, different research groups and companies have studied air quality, provided new innovative solutions and made full use of the various technologies available. A very important example is provided by the information giant Google, which has recently integrated the possibility of displaying the atmospheric status from its map localization app Google Maps.

In this context, the O-ZONE project was born; some students of the Aerospace Engineering course at the University of Padova at the beginning of 2019 engaged in the design and development of alternative sampling systems [5]. Currently, measurements of atmospheric pollutants are mainly carried out by satellite (ESA’s Sentinel Mission [6]) and by ground using static samplers. The study of the atmosphere in situ employing systems that can estimate the atmospheric composition at different altitudes and identify pollutants to understand how they can interact and then spread over time remains an open field.

With the support of Professors Alessandro Francesconi and Roberta Bertani, the students then applied to participate, with their experiment, in the BEXUS project of SNSA, DLR and ESA [7]. The acceptance of O-ZONE then led the team to launch on 30 September 2021, at the Esrange base in Kiruna, the prototype for the first test collecting atmospheric data [8]. It is highlighted that the data collected is of high scientific interest following the COVID-19 pandemic that altered atmospheric composition in 2020 and 2021 [9]. The coronavirus also allowed the O-ZONE team to improve the initial prototype making the launch, delayed due to the pandemic, obtain better results.

This paper briefly describes the design of O-ZONE, the flight aboard the BEXUS 30 balloon, the data collected and the conclusions based on the interpretation of these data.

## O-ZONE Payload

O-ZONE is an atmospheric air sampler designed to operate at different altitudes and collect various pollutants, especially VOCs (Volatile Organic Compounds) and, below 5000 m, PM (Particulate Matter). The samples collected by the payload are then analysed on the ground to reduce the costs associated with in situ analysis. The device underwent two design phases, with the first design being revised to enable higher reliability and facilitate reuse.

The O-ZONE experiment can be divided into three main subsystems: pneumatic, chemical sampling and control [8]. There are three sampling components: the VOC filter, the PM filter and the collection bag. In the current configuration, there is only one PM filter, as a single control sampling is carried out within an altitude of 5 km, whilst there are four VOC filters to allow sampling at various altitudes.

**Fig. 1:**
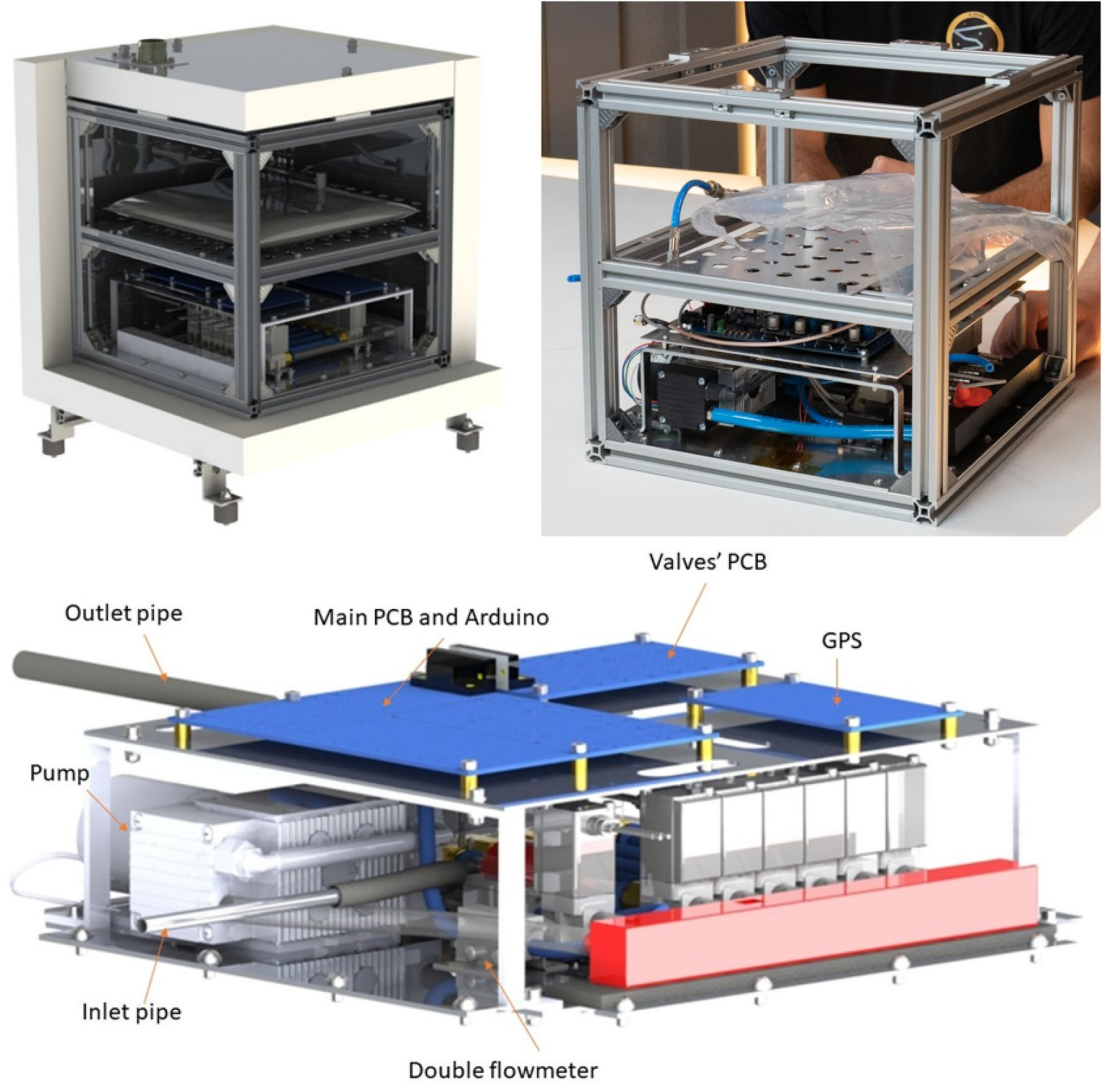
Setup of the O-ZONE experiment; top left full view, right a photo of the system without insulation, bottom zoom on the pneumatic and electronic subsystems

The 3 systems are closely arranged in different compartments and are housed in an aluminium frame made of BOSCH profiles, as can be seen in Fig. 1. The box is shielded on each side by multi-layer panels of polystyrene and aluminium (with a structural and protective function), with an additional aluminium foil on the outside to reflect the sun's rays.

O-ZONE is fixed to the gondola employing rubber bumpers, which prevent current flows and dampen vibrations and eventual shocks (such as the one that occurs during a controlled fall).

**Fig. 2:**
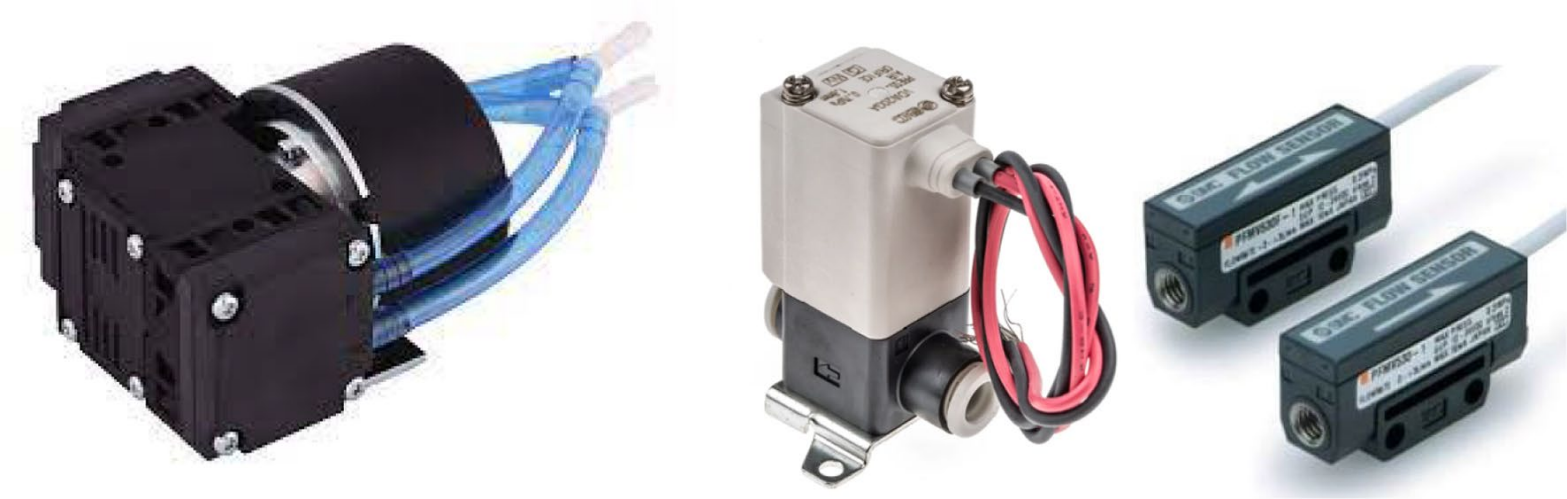
Main components of the pneumatic subsystem; starting from the left, N85.3 KPDCB pump by KNF, VDW20QAP valve and PFMV5 flowmeters both from SMC. (Photo credits to KNF and SMC)

The design of the pneumatic system starts with the pump, the real heart of the experiment, which allows the suction of the external air. This four-wire pump is a KNF (model N85.3 KPDCB in Fig. 2) [10], a diaphragm pump that was chosen mainly because of the low contamination rate of the air collected; any other type of pump could have distorted the analyses by contaminating the air with oil or other lubricating products.

**Fig. 3:**
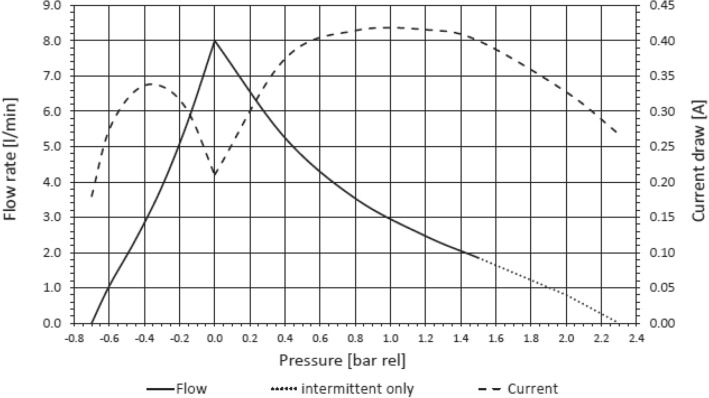
Pump performance in relation to pressure from KNF data sheet

**Fig. 4:**
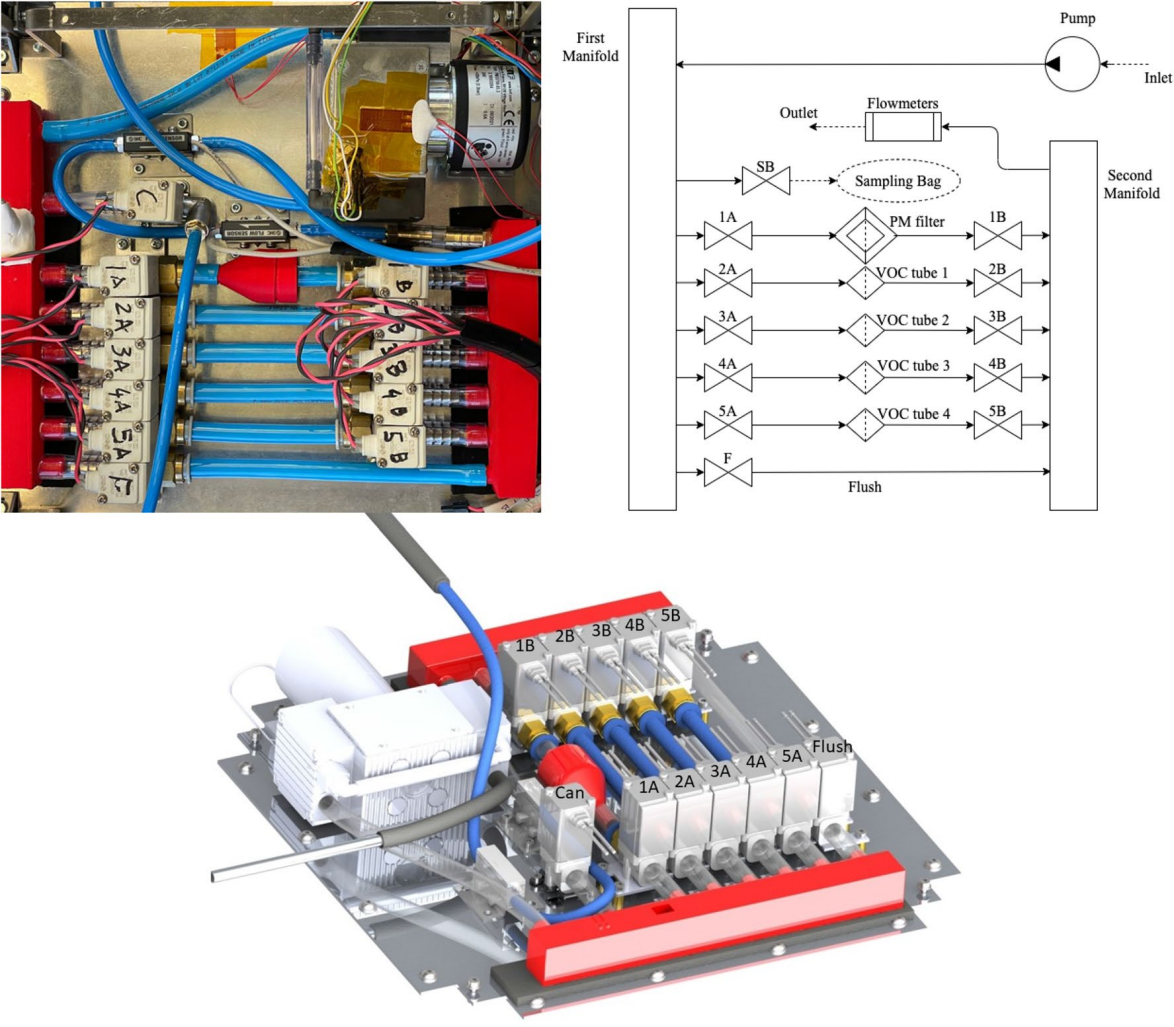
Overview of the pneumatic subsystem; top picture and diagram, bottom CAD reproduction

Another reason for choosing this pump is the head that is typical of this type of pump, which is designed for vacuum (Fig. 3). The air flows through the pump, located upstream of the pneumatic system, to reach the first manifold which has, as can be seen in Figs. 4, 7 possible outings, one for each of the filters (4 VOC and 1 PM), one for the sampling bag and one for the route used to clean the pneumatic system. Each way is regulated by a pair of solenoid valves (SMC model VDW20QAP) [11], which guarantees an additional safety net as if the system should fail to respond, they stay sealed, preventing the samples from getting contaminated. After passing through the various ducts, these re-join in a second manifold that ends with the outlet pipe. Before exiting, the air passes through two flow metres, both same model (PFMV5 from SMC) but with different scales [12]. This way it is possible to obtain accurate measurements for both the airflow rates needed during the flight, which is necessary to meet the sampling requirements of the filters and to keep the flow rate constant compensating for the effects caused by the variations in pressure.

The pump is controlled by the Arduino which reads the flow rate data. The pipes have an internal diameter of 6 mm except for those between the pairs of valves which have an internal diameter of 8 mm to allow the housing of the VOC filters. The particulate filter holder and collectors were designed by the team and 3D printed in plastic to reduce weight and better fit into the limited space.

The high number of joints and systems involved may cause pressure losses, so various tests have been carried out in a vacuum chamber at various pressures. This way, knowing the output flow rate and the external pressure, it is possible to trace the flow rate injected into the system just before the filters.

**Fig. 5:**
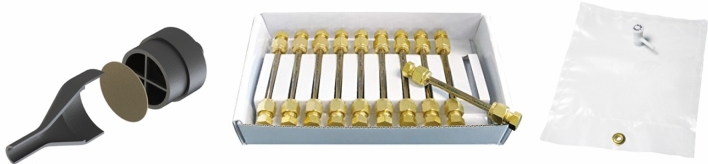
Elements of the sampling system; starting from the left PM filter, desorption tubes (Photo by RESTEK) and sampling bag (Photo by RESTEK)

The sampling system, as mentioned before, consists of filters and the sampling bag (Fig. 5). The sampling bag is located on the top shelf of the device and has a maximum capacity of 3 L. To ensure that the sampling bag is empty before the flight, the pipe connecting it to the valve at the exit of the manifold is fitted with an at-joint ending in a non-return valve, creating a vacuum and ensuring that no air from the wrong altitude contaminates the sample. Desorption tubes were used for VOCs and quartz filters for PM. The choice of filters aims to involve a broad spectrum of combustion products but also takes into account their reusability, which makes O-ZONE a sustainable experiment from all points of view. The proposed experimental configuration may vary depending on the gondola and the analytes being researched.

**Fig. 6:**
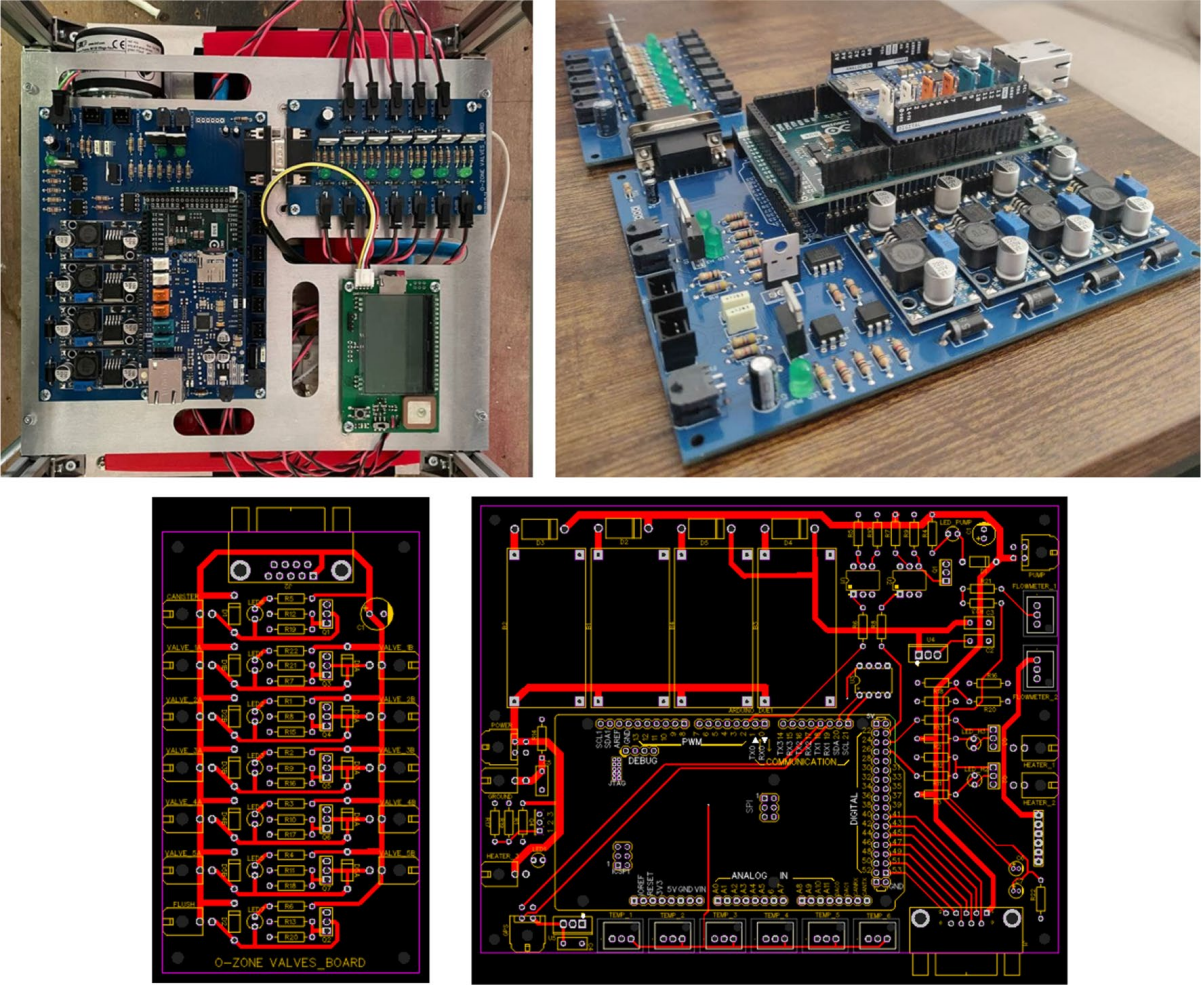
Top two shots of the PCBs, bottom the design schematics

Regarding the control system, O-ZONE is powered by a voltage of 28.8 Volts and an average current of 1.3 Ampere, which was provided, for the flight in Kiruna, by an external battery pack. Also, the antenna and its Ethernet connexion with the experiment are possible, thanks to the gondola’s bus. Although the experiment could operate autonomously, this redundancy proved useful in the test phase to allow a real-time verification of housekeeping and device operation. The onboard computer is an Arduino Due, with an Ethernet shield, connected to the PCB designed by the team (Fig. 6). It switches on and off the pump and the valves, and in the meantime receiving the data from the GPS and the temperature and pressure sensors.

This way, O-ZONE is completely autonomous and, by interpreting altitude data, manages the correct order of the filters at various atmospheric layers. An additional duty of the control system is to check that the thermal ranges of the various components are respected. In fact, in addition to the passive thermal control system, which consists of the polystyrene box described above, O-ZONE is equipped with an active system composed of two heaters, housed on the base plate, which increase the temperature of the experiment when necessary.

**Fig. 7:**
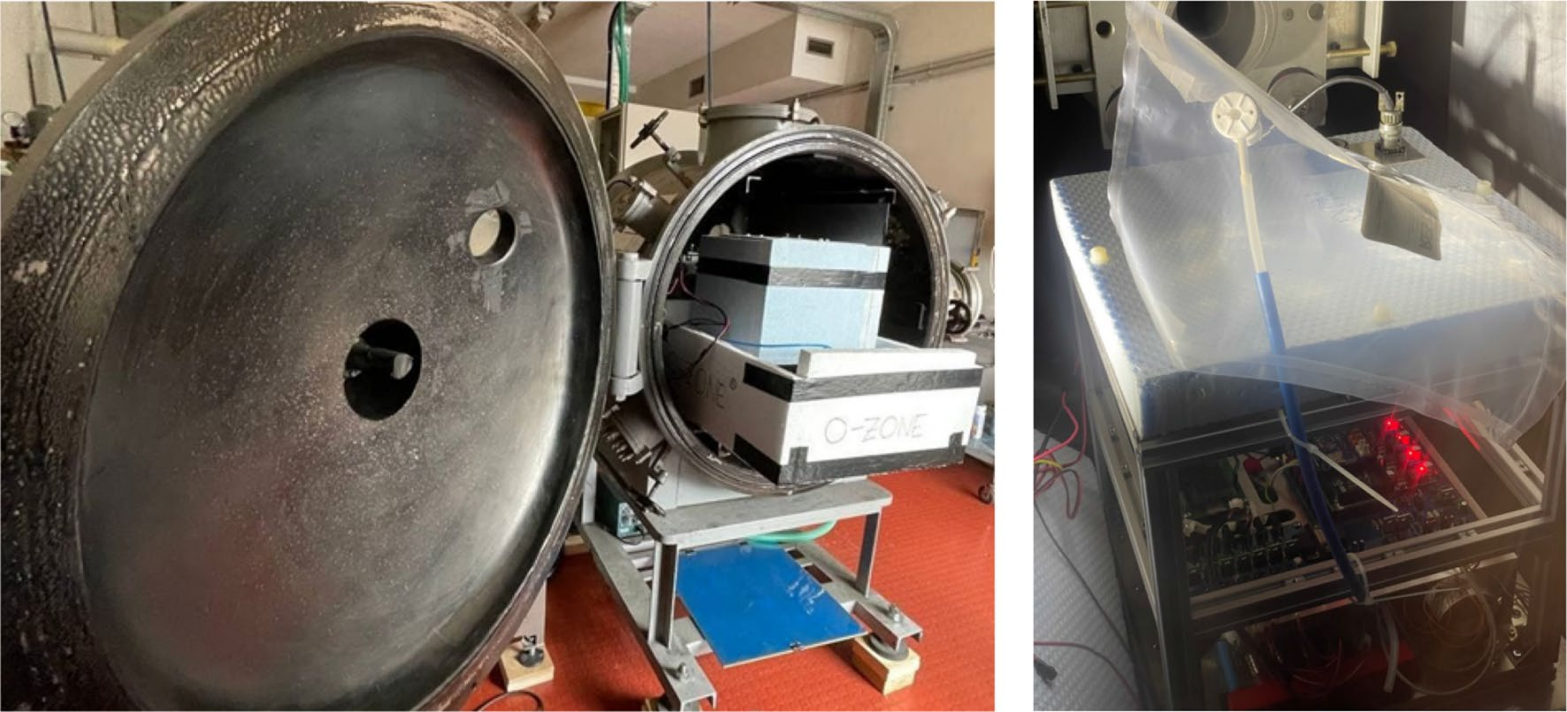
Thermo-vacuum text in CISAS G. Colombo centre facilities

**Fig. 8:**
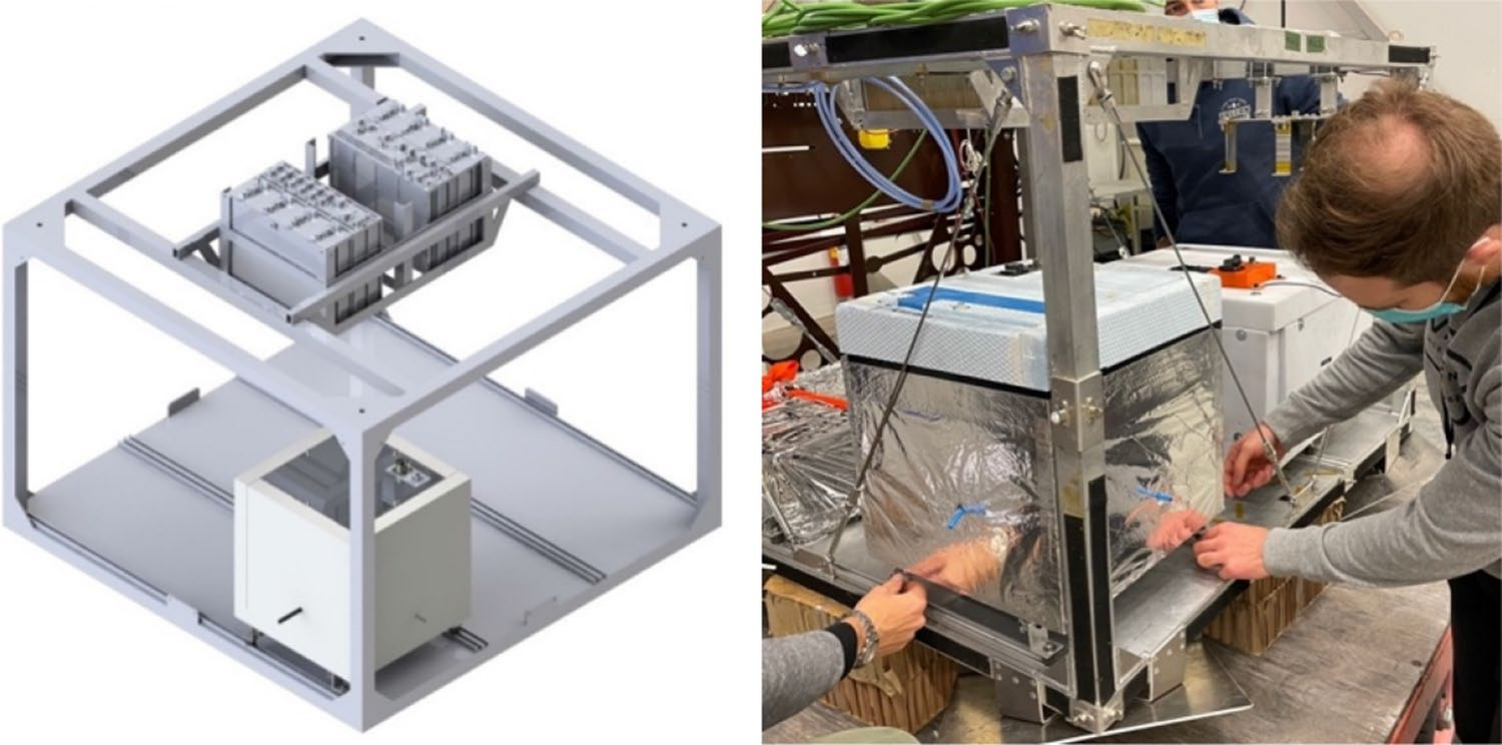
Location of the O-ZONE experiment on the SSC gondola

After the assembly phase, the team began the testing phase. Considering that the minimum pressure reached by the device would be around 10^−2^ bar and the minimum temperature would be – 70 °C (at an altitude of 30 km [13]), it was appropriate to test O-ZONE in a thermo-vacuum chamber. However, the cleanliness of the experiment only met the characteristics of the vacuum chamber and not the thermo-vacuum of the available facility (CISAS “G. Colombo”, the space centre of the University of Padova). To implement the thermal test as well, the following approach was therefore taken: a conductive metal interface, known as a “cold plate”, was attached to the experiment’s fastenings (Fig. 7), this simulated the gondola which, during flight, would be the only conductive element in contact with the inner frame of O-ZONE (Fig. 8). This plate was placed in direct contact with dry ice and, although this releases CO_2_, the chamber was kept between 10 and 15 mbar. This set-up allowed an extensive test of the experiment under realistic conditions that were confirmed after the mission.

The described device flew onboard the BEXUS 30 after the complete verification of the mentioned requirements. All the operational phases of the flight are described in the next chapter. 

## Flight on BEXUS 30

The flight aboard the BEXUS 30 gondola (Fig. 8) took place at the SSC (Sweden Space Corporation) base in Kiruna, called Esrange. As per the BEXUS project of SNSA, DLR and ESA [8], many experiments are on board the gondola. In our case, they were all experiments related to chemical and biological research, which was positive in many aspects, amongst them the comparison of data.

**Fig. 9:**
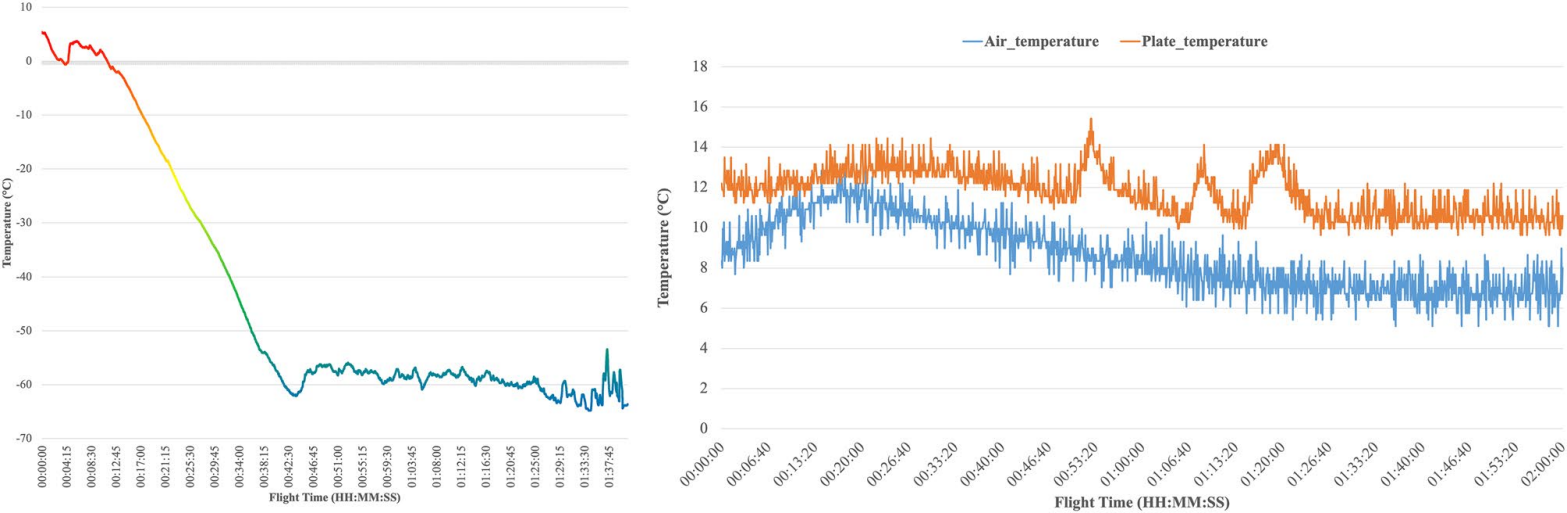
Temperature data during the flight; on the left the external temperature, on the right the internal ones

At 06:54 local time on 30 September 2021, O-ZONE, together with the other devices, began its flight aboard the gondola weighing approximately 120 kg in total. The ascent phase, which lasted approximately 2 h, marked the end of the filter-sampling phase and the start of the sampling bag phase. The bag was completely inflated a few minutes after reaching the maximum altitude of 27.3 km; the floating phase lasted approximately 3 h and the experiment was kept in stand-by with controlled temperature, as shown in Fig. 9, to preserve the samples and avoid damage to the electronic subsystem.

During the first 5 km, the airflow was directed into the PM filter, and its rate was around 3 L/min as per the filter’s requirement. Subsequently, the air was conveyed towards the VOC filters. The first VOC filter sampled the air between 5 and 9.5 km, the second between 9.5 and 14 km, the third between 14 and 18.5 km, the fourth and last filter between 18.5 and 23 km. Finally, during the floating phase, the air was conveyed into the sampling bag which was filled with approximately 1 L of air after 30 min of intake.

The filters just described have quite strict flow rate requirements to carry out a correct sampling and therefore the pump was regulated by PWM to guarantee a flow rate of about 0.05 L/min. In the table below (Table 1), a summary of all the phases can be seen, with the durations, altitudes, temperatures and flow measurements taken.

**Table 1:** O-ZONE flight data

Phase	Start time [hh:mm:ss]	End time [hh:mm:ss]	Altitude range [m]	External pressure [mbar]	*Average internal flow rate [L/min]	Inside temperature [°C]	External temperature [°C]
Cleaning tube	06:55:00	06:58:00	500	962.4	Variable	12.0	4.0
PM tube	06:58:00	07:13:00	5000	546.2	2.933	12.5	− 14.8
Cleaning tube	07:13:35	07:15:30	5500	511.1	Variable	13.0	− 18.5
First VOC tube	07:15:30	07:29:50	9500	289.1	0.049	13.5	− 49.0
Cleaning tube	07:30:00	07:31:00	10,000	267.6	Variable	12.2	− 53.3
Second VOC tube	07:31:30	07:46:30	14,000	141.9	0.048	13.0	− 56.2
Cleaning tube	07:47:20	07:48:13	14,500	131.1	Variable	13.2	− 57.9
Third VOC tube	07:48:23	08:03:23	18,500	69.4	0.048	13.0	− 58.6
Cleaning tube	08:03:54	08:05:23	19,000	64.1	Variable	11.0	− 57.6
Fourth VOC tube	08:05:23	08:20:23	24,100	28.4	0.048	9.9	− 60.8
Cleaning tube	08:21:10	08:22:10	25,000	24.5	Variable	9.9	− 63.0
Sampling bag	08:22:18	08:52:18	27,000	17.7	0.029	10.0	− 61.0

*The cleaning phase was used not only to clean the tubes between each phase, but also to set the correct flow rate

Once the floating phase was over, the gondola began its controlled fall, landing in Finland at 11:33 local time after 5 h of total flight time. Recovery took place in the following 24 h and the collected samples were taken to the laboratory for analysis.

In the next chapter, the sample analysis methodologies and related results are described.

## Data and Analysis

The flight aboard the BEXUS 30 gondola enabled to collect data at different altitudes, namely 4.5, 9, 13.5, 18, 22.5 and 25 km. Volatile organic compounds (VOC), chlorofluorocarbons (CFC) and particulate matter (PM) were collected and analysed.

Volatile organic compounds (VOC) and chlorofluorocarbons (CFC) were collected using a constant flowrate of 0.05 L/min, which was stated within the performance requirements. The sampling was performed using four Tenax TA/Graphitized Carbon Black/Carboxen 1000 stainless steel TD tubes. After the experiment, the latter taken out, sealed and afterwards placed in an isothermal box at 4 °C for 4 days. This temperature was chosen to prevent potential migration of chemicals to more retentive sorbent. Indeed, this may lead to incorrect concentrations of the analytes. Samples were analysed by SGS Italia S. p. A. in compliance with the method of analysis NIOSH 2549. VOCs were also sampled with a sampling bag made by polyvinylidene fluoride (PVDF), volume of 3 L. The sampled materials were analysed by SGS Italia S.p.A. employing the methods EPA 3C 2017 and EPA TO 15 1999.

Particulate matter (PM) was collected with PM homemade filter system made by two filters, both placed in the same slot of the device. The first was a Whatman^®^ QM-A quartz filter that trapped the PM via inertial impact. This microfiber filter was made by a disc of microporous quartz (SiO_2_) and it had an average pore size of 2.2 µm. The second filter was a microporous polycarbonate filter, which was a membrane composed by high-quality polycarbonate film with a sharply defined pore size of 0.8 µm diameter.

The characterization techniques employed were standard analyses for these air pollutants. The methods used were Fourier-transform infrared spectroscopy (FTIR), environmental scanning electron microscopy (ESEM) and chromatography–mass spectrometry (GC–MS).

The VOCs and CFCs were collected at different altitudes during the flight. Instead, the atmospheric PM sampling was carried out in the first part of the ascending phase, so at low altitudes. If the measurements were performed over a longer period, they would provide more data and information on concentrations. The values reported herein are the result of preliminary tests which were carried out to verify the performance of the device before and after the launch campaign.

The experimental conditions at which the VOCs samplings were performed in Kiruna are reported in Table 2, whilst those for PM are shown in Table 3.

**Table 2:** Conditions under which sampling, through VOC filters and sampling bag, was carried out during the launch campaign

	VOC 1	VOC 2	VOC 3	VOC 4	Sampling bag
Time [s]	855	855	855	855	1780
Litres [L]	0.7075	0.7225	0.7225	0.7225	0.8820
Temperature [°C]	10.58	9.38	8.16	7.29	6.89
Humidity [%]	1.6	0.4	0.4	0.4	0.4
Initial pressure [mbar]	513.39	270.3	132.56	62.88	26.86
Final pressure [mbar]	290.85	144.32	68.57	29.98	17.54

**Table 3:** Conditions under which sampling, through PM filters, was carried out during the launch campaign

Time [s]	890
Litres [L]	43.5
Temperature [°C]	10.78357542
Humidity [%]	13.45586592
Initial pressure [mbar]	876.67
Final pressure [mbar]	560.27

The most significative concentrations of VOCs collected during the lunch campaign in Kiruna are reported in Tables 4 and 5.

**Table 4:** Results of the chemical analyses conducted on VOC filters

Sampled volume [m^3^]		2.97 × 10^−4^	1.59 × 10^−4^	7.8 × 10^−5^	3.6 × 10^−5^
Flow rate [L/min]		0.05	0.05	0.05	0.05
Compound	Method	Value VOC 1 [µg/m^3^]	Value VOC 2 [µg/m^3^]	Value VOC 3 [µg/m^3^]	Value VOC 4 [µg/m^3^]
2-methylpentane	NIOSH 2549 1996	67.34	283.02	< 256.41	< 555.56
3-ethylpentane	NIOSH 2549 1996	114.48	345.91	< 256.41	< 555.56
Benzene	NIOSH 2549 1996	181.82	245.28	512.82	2500.00
Cyclohexane	NIOSH 2549 1996	101.01	< 125.79	< 256.41	< 555.56
n-Dodecane	NIOSH 2549 1996	215.49	301.89	371.79	1333.33
Ethyl benzene	NIOSH 2549 1996	144.78	213.84	< 256.41	< 555.56
Meta + para xylene	NIOSH 2549 1996	205.39	295.60	< 256.41	1166.67
1-butanol	NIOSH 2549 1996	2962.96	4088.05	< 256.41	4611.11
Orto-xylene	NIOSH 2549 1996	148.15	207.55	< 256.41	< 555.56
Styrene	NIOSH 2549 1996	309.76	440.25	< 256.41	944.44
Toluene	NIOSH 2549 1996	185.19	232.70	< 256.41	972.22

**Table 5:** Results of the chemical analyses conducted on a sample of air (sampling bag)

Compound	Method	Value [µg/m^3^]	Compound	Method	Value [µg/m^3^]
1,2,3-trimethylbenzene	EPA TO 15 1999	0.46	Cyclohexane	EPA TO 15 1999	15.3
1,2,4-trimethylbenzene	EPA TO 15 1999	1.80	Dichlorodifluoromethane	EPA TO 15 1999	3.09
1,2,5-trimethylbenzene	EPA TO 15 1999	0.51	Chloromethane	EPA TO 15 1999	1.80
4-ethyltoluene	EPA TO 15 1999	0.50	Carbon disulfide	EPA TO 15 1999	2.53
2-propanol	EPA TO 15 1999	920	Dichloromethane	EPA TO 15 1999	3.38
Acetone	EPA TO 15 1999	77	Ethanol	EPA TO 15 1999	510
Acrylonitrile	EPA TO 15 1999	2.53	Ethyl benzene	EPA TO 15 1999	2.14
Acrolein	EPA TO 15 1999	2.83	Hydrocarbon C5-C12	EPA TO 15 1999	124
Trichlorofluoromethane	EPA TO 15 1999	1.51	Styrene	EPA TO 15 1999	5.1
Methyl acetate	EPA TO 15 1999	2.28	Naphthalene	EPA TO 15 1999	1.28

**Fig. 10:**
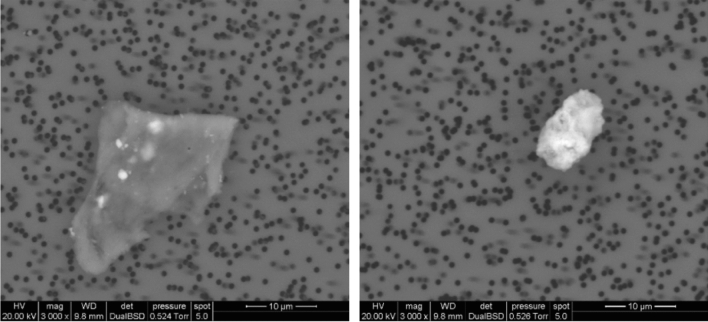
ESEM image of polycarbonate filter, on left an organic particle is displayed whilst an inorganic particle is shown on the right

**Fig. 11:**
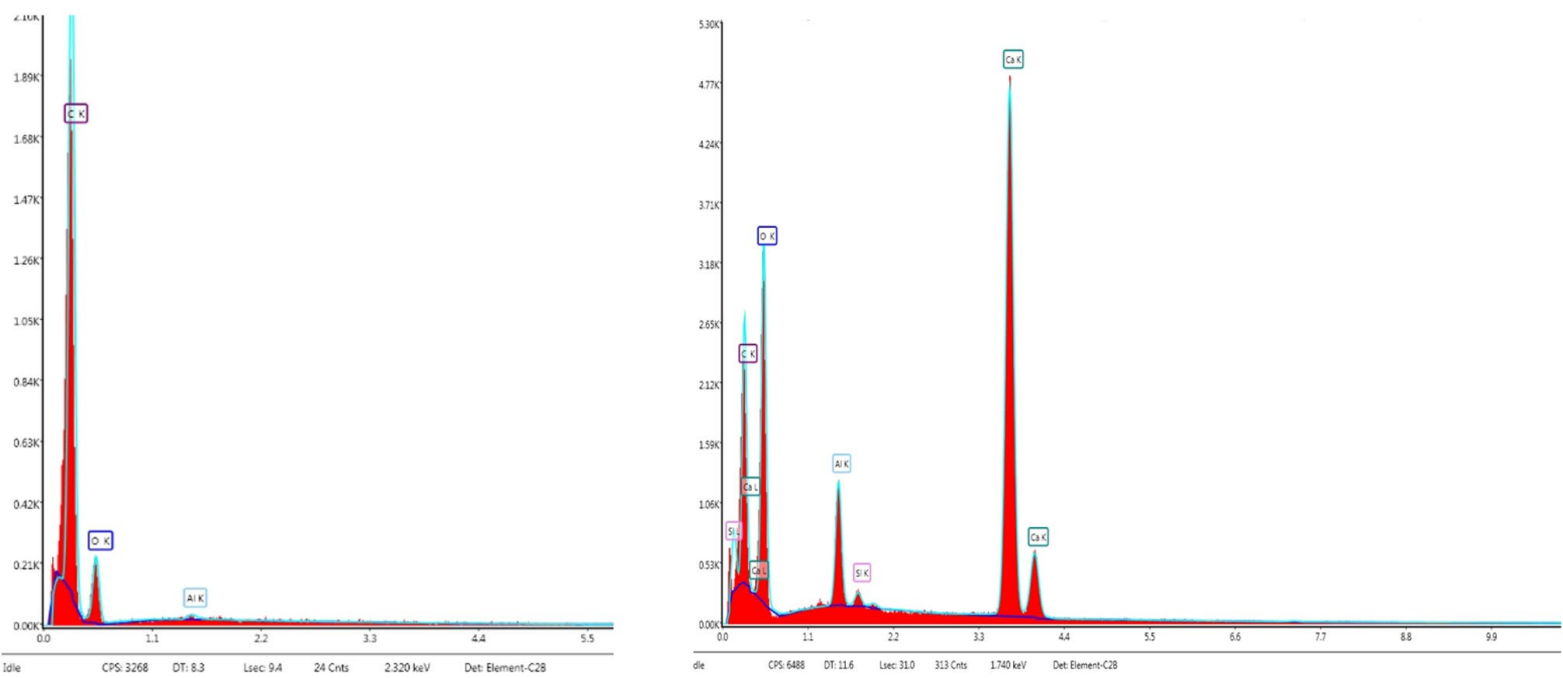
EDX spectra. On the left of an organic Polymer made of C and O, on the right of inorganic particle containing CaO, Al_2_O_3_, SiO_2_

The PM filters were qualitatively investigated by ESEM (Fig. 10). Although no analytes of any kind could be observed on the first Quartz disc, the polycarbonate filter collected few both organic and inorganic particles with dimensions ranging from 5 to 10 μm. It was possible to identify the composition of those particles using EDX analysis (Fig. 11). In Fig. 10, the lighter and brighter spots stand for the inorganic components (mainly Silicon, Calcium and aluminium) whilst the transparent particle represents an organic compound.

From a chemical point of view, the prototype proved to be operating [14, 15]. The chemical analyses revealed the presence of many compounds belonging to the class of chlorofluorocarbons, aliphatic and aromatic hydrocarbons and other VOCs.

The system was able, at all assigned altitudes, to sample all the analytes of interest (VOCs, CFCs and PM) but also to provide a complete picture of the compounds present in the air with concentrations in the order of µg/m^3^. The data are here reported (Tables 4 and 5) without any error because so far, they are not enough to either validate the method or to estimate the sampling error [14]. The experiment should be repeated other times to verify the reliability and integrity of this promising and low-cost sampling tool.

The main problem encountered in this flight was the presence of undesired chemicals released from other experiments in the BEXUS 30 gondola. In fact, the presence of other devices on board the gondola and the choice of some of the materials, especially insulating ones, led to undesirable outgassing of alcoholic compounds and the presence of chemical species not in agreement with those found in the literature [13].

This obviously biassed the sampling of some chemicals, whose concentrations were much higher than expected or showed an irregular pattern. Nevertheless, the device was able to collect many chemical substances and allowed to qualitatively estimate their concentrations. This confirmed the expected functional and design requirements and opened a window for quantitative estimates. Certainly, this must be proven by other dedicated launches where the experiment is not affected by other devices or materials that may alter the normal chemical composition of the analysed air, otherwise leading to an overestimation of concentrations.

Therefore, it is possible to further optimise this already promising device and then confirm data already known to the scientific community [16]. However, to make a consistent comparison between literature and chemicals collected by O-ZONE, sampling data or quantitative measurements of the same compounds sampled at the same time and in the same areas are required—both with regard to latitude and longitude but also with regard to altitude. This was not possible because of the variability of pollutants due to the historical period (Coronavirus pandemic [9]) but also due to the low frequency of data collection in those areas, which demonstrates the usefulness of the device designed as a tool for sampling areas under low surveillance but of high importance.

In fact, as described in the literature [17], more inert pollutants such as CFCs are distributed in the polar regions of the globe, making large-scale sampling and measurements in the upper atmosphere increasingly necessary.

## Conclusions and Future Uses

In conclusion, O-ZONE proved to be an effective device for dynamic sampling at various altitudes. By refining the choice of the quantities of air sucked in, the atmospheric composition can be characterised more clearly. The ideal set-up of the payload should aim to avoid any interference between different devices.

Some of the chemical species found were produced onboard the gondola by outgassing of O-ZONE materials and other devices [18]. Therefore, calibration by adding a degassing phase to detect and eject unwanted chemical species should be made before the flight. The functioning of the device has been demonstrated even at high altitudes in complete autonomy, and this guarantees safety for future flights and confirms the choice of critical components, such as the pump, valves and onboard computer.

In situ sampling and ex situ analyses comported better reliability, avoiding the implementation of systems with hard to fulfil at high altitudes requirements. Economically speaking, the prototype proved to be very affordable given that most of its components are easy to get, low cost and reusable.

As anticipated, the results cannot be confirmed from a quantitative point of view as similar analyses are few in number and do not examine the same atmospheric stages considered during flight. However, from a purely qualitative point of view, it can be stated that, barring a few anomalous species whose presence has been justified (e.g. other experiments on board the gondola), there are no other compounds that could invalidate the analyses. This makes understandable the need for devices that fill the analysis gap left by satellites and ground bases both for better spatial resolution and for reaching the areas of interest.

The potential applications of O-ZONE are enhanced by its ability to sample many VOCs simultaneously, as the sampling bag results table shows (Table 5). Additionally, the ability to collect pollutants of interest, such as VOCs, CFCs and PM, at all assigned altitudes was demonstrated during the flight (Table 4). Furthermore, considering the high variety of pollutant sources, tools such as the one developed can improve large-scale environmental monitoring by identifying natural and man-made sources of the main climate change actors.

This instrument is open to different possible future uses: aside from its original monitoring nature, the device could also be used in a horizontal way instead of the BEXUS ascending flight. This way, it would be easy to obtain data on phenomena area of effect. One example could be finding out how far from a volcano its ashes are still present in the air at a certain percentage. The set-up can also vary and be made lighter depending on the environmental requirements and the host vehicle, e.g. the device could be on top of drones for monitoring crops or industries. A future development could precisely be the monitoring of agricultural crops for which standards are difficult to verify and often lead to scandals and investigations concerning organic certification [19].

## Data Availability

All data collected by the O-ZONE experiment are freely accessible and available in the experiment's technical document [20].
